# Prevalence of anemia and associated factors in a Moroccan population from the Northwestern region of Morocco (M’diq-Fnideq-Martil Prefecture)

**DOI:** 10.11604/pamj.2023.44.131.35991

**Published:** 2023-03-15

**Authors:** Saad Bakrim, Najoua El Hichou, El Khalil Ben Driss, Sara Aboulaghras, Abdelaali Balahbib, Abdelhakim Bouyahya, Azlarab Masrar

**Affiliations:** 1 Geo-Bio-Environment Engineering and Innovation Laboratory, Molecular Engineering, Biotechnology and Innovation Team, Polydisciplinary Faculty of Taroudant, Ibn Zohr University, Agadir, Morocco,; 2 Hematology Laboratory, Faculty of Medicine and Pharmacy, Mohammed V University, Central Hematology Laboratory, Ibn Sina University Hospital, Rabat, Morocco,; 3 Department of Biology, Faculty of Sciences, Abdelmalek Essaâdi University, Tetouan, Morocco,; 4 Physiology and Physiopathology Team, Faculty of Sciences, Genomic of Human Pathologies Research, Mohammed V University in Rabat, Rabat, Morocco,; 5 Laboratory of Biodiversity, Ecology and Genome, Faculty of Sciences, Mohammed V University, Rabat 10106, Rabat, Morocco,; 6 Laboratory of Human Pathologies Biology, Department of Biology, Faculty of Sciences, Mohammed V University in Rabat, Rabat, Morocco

**Keywords:** Anemia, school children, pregnant women, sociodemographic status, food consumption, northwestern, Morocco

## Abstract

**Introduction:**

anemia remains a major public health challenge worldwide, frequently having multifactorial causes and wide-ranging, largely underestimated repercussions. The purpose of this paper is to assess the prevalence of anemia and identify associated factors in a group of children, adults, and pregnant women.

**Methods:**

our sample consisted of a total of 1360 volunteers (group I: 410 school-aged children aged 5-11 years; group II: 533 adults aged 16 to 65 years; group III: 417 pregnant women aged 17 to 45 years) randomly selected from different towns of the M'diq-Fnideq prefecture, Morocco from March 2018 to September 2018. Data on socio-demographic, anthropometric, and dietary status were collected from a questionnaire survey. A complete blood count was performed using a hematology analyzer, Sysmex KX21N® (Sysmex Corporation, Kobe, Japan), in the hematology laboratory of the Mohamed VI Hospital of M'diq.

**Results:**

anemia was found in 31% of children, 52.4% of adults, and 22.5% of pregnant women. Microcytic hypochromic anemia was the most dominant type of anemia in children, adults, and pregnant women with percentages of 40.6%, 48.7%, and 43.5%, respectively. Mild anemia was much more common than moderate and severe anemia in all groups. Furthermore, anemia was associated with low socioeconomic and educational levels in adults (22.8% versus 27.9%) and pregnant women (18.1% versus 16.8%). Schoolchildren with illiterate parents and low socioeconomic levels are the most affected by anemia, with a prevalence of 75% and 69.44%, respectively. Also, children with insufficient stature are at a high risk for anemia compared to children of normal stature (p<0.001). As for weight for age, the odds ratio (OR) was 4.32. A significant difference between underweight and anemia was revealed (p<0.001). A frequency of meat product, vegetables, and fruit consumption lower than 1.5 times per week increases the risk of anemia in schoolchildren.

**Conclusion:**

these findings showed a significant prevalence of anemia in all study groups associated with socioeconomic, anthropometric, and nutritional factors. However, further studies are needed to focus on interventions and etiologies in order to limit potential complications, especially in schoolchildren and pregnant women.

## Introduction

Anemia continues to be one of the most prevalent public health challenges, affecting both developed and developing countries. It has serious repercussions on health and well-being, as well as social and economic implications. Indeed, although living conditions have improved remarkably in recent decades, anemia affects physical growth, cognitive development, reproduction, and physical work capacity, among other things, resulting in decreased human performance [1]. According to the World Health Organization (WHO), anemia is defined as a decrease in the concentration of hemoglobin (Hb) below the limit values in relation to the age, sex, and physiological status of individuals [2]. It is widespread among all age categories and is the most frequent manifestation of micronutrient deficiency worldwide [3]. Southeast Asia has the highest overall prevalence of anemia in preschool children with a prevalence of 60% [4]. The most common cause of anemia in the world is iron deficiency. Developing countries have the highest prevalence with 60% in pregnant women, 50% in children under 4 years of age, and 45% in school children [4]. The Moroccan population is not excluded from this condition, since it is affected by dietary deficiencies resulting from, among other factors, changes in lifestyles and eating habits. At the national level, iron deficiency anemia touches more than a third of the Moroccan population with a predominance of pregnant women (37.2%), women of childbearing age (33%), children aged 6 months to 5 years (31.6%), and finally men (18%) [5]. Considering the significance of this disease issue, which is acknowledged as a public health problem, little progress has been reported in this area. In the M'diq-Fnideq prefecture in northwestern Morocco, there is limited published information on the prevalence of anemia. In order to address this gap, we proposed to conduct a study to estimate the prevalence and determine the factors associated with anemia in a population composed of adults, pregnant women, and schoolchildren living in the prefecture of M'diq-Fnideq, northwestern Morocco. The purpose is to promote effective prevention and implementation of appropriate health programs to manage the risks of anemia.

## Methods

**Site of the study**: the Prefecture of M'diq-Fnideq, created in 2004, is a predominantly urban subdivision of the Tangier-Tetouan-Al Houceima region (geographical coordinates 35.7^º^N 5.4^º^W). It covers an area of approximately 178 km^2^. It is located in the extreme north of the Kingdom of Morocco, and bounded on the north and east by the Mediterranean Sea, south by the province of Tetouan, and west by the province of Fahs-Anjra. Its population was established, according to the results of the general census of population and housing of 2014, with 209.897 inhabitants, representing 5.9% of the population of the region Tangier-Tetouan-Al Hoceima and 0.6% of the national population. As for the average density, it is about 538 inhabitants/km^2^. The prefecture of M'diq-Fnideq is characterized by a Mediterranean climate with relatively low-temperature fluctuations. Five cities are located there: Martil, Fnideq, M'diq, Alliyenne, and Belyounech.

**Type of study**: this is a descriptive and analytical cross-sectional study that was conducted at Mohammed VI Hospital in M'diq during the period from March 2018 to September 2018.

**Study population**: our sample consisted of a total of 1360 volunteers randomly selected from the hospital's outpatients, hospital staff, companions and visitors living in the different towns of the M'diq-Fnideq prefecture. They were classified into three groups: a) **Group I**: 410 school-aged children aged 5-11 years; b) **Group II**: 533 adults aged 16 to 65 years; c) **Group III**: 417 pregnant women aged 17 to 45 years.

**Inclusion and exclusion criteria**: we initially included 1360 subjects willing to participate voluntarily in the study, belonging to different socio-professional categories and coming from different cities of the prefecture: M'diq, Fnideq, Martil, Alliyenne, and Belyounech. All individuals with hematological, digestive, and inflammatory complications and those who were not part of the prefecture studied were excluded from the study. All observations were made by a medical team from the medical department of the Mohammed VI Hospital in M'diq. By adopting these criteria, only 1285 subjects were finally retained for the study of which: group I: 385 schoolchildren, group II: 513 adults, and group III: 387 pregnant women. The latter performed a blood count at the medical biology department of the Mohammed VI Hospital in M'diq during the study period.

**Data collection: Group I**: the following data were collected from a questionnaire survey completed by parents; a) children's identity: age, gender, and place of residence; b) socioeconomic status was defined by the following parameters: educational level and socioeconomic level of parents; c) anthropometric measurement was based on the WHO and United Nations Children's Fund standard method.

Children's weight was measured to the nearest 0.1 kilograms on a new mechanical scale with an accuracy of 0.5 kg. Height was measured to the nearest 0.1 cm on a bar and steel moving-belt scale. Height for age, weight for age, and weight for height were determined by Z-points and calculated with Epinfo. Underweight, wasting, and height deficiency were defined by WHO for Z-points of height-for-age, weight-for-age, and weight-for-height below -2. Biological data: blood count (Hb level, mean corpuscular volume (MCV) and mean corpuscular hemoglobin concentration (MCHC)).

**Group II**: the adult group completed a questionnaire with the following information: a) participant identity: age, gender, and place of residence; b) socio-demographic parameters: education level and socioeconomic status; c) sociological data: blood count (Hb level, MCV, and MCHC).

**Group III**: pregnant women completed a questionnaire prior to each blood draw. The following information was sought: a) the identity of pregnant women: age, age of pregnancy and place of residence; b) socio-demographic data: education level and socioeconomic status; c) biological data: blood count (Hb level, MCV and MCHC).

**Blood collection**: for each participant, blood samples were collected in the morning, at the elbow crease, in 5ml BD Vacutainer^®^ system tubes (13×75 mm) containing Tri-potassium Ethyl Diamine Tetra Acetic (K3EDTA) as an anticoagulant. Blood counts were performed on the same day within 2 hours post-sampling.

**Hematology analysis**: the haemogram was performed by a hematological analyzer of the Sysmex KX21N^®^type (Sysmex Corporation, Kobe, Japan) in the hematology laboratory of the Mohamed VI Hospital of M'diq. The KX-21N^®^ processes approximately 60 samples per hour and allows to specify 19 blood parameters. The information collected is red blood cell (RBC) count, hematocrit (Hte), Hb, MCHC, and MCH. For each series of analyses, the validity of the measurements is ensured by running three control samples: normal control blood, low pathological control blood, and a third high one. The results obtained from these samples must fall within the range of values provided by the manufacturer for the control samples.

**Operational definitions**: to make the biological diagnosis of anemia, we opted for the Hb cut-off values established by the WHO defining anemia and its severity. To study the typical profile of anemia, we used the cut-off values of MCV and MCHC used by the prefecture physicians to interpret the blood count values.

The cut-off values considered were as follows: **Group I (school-aged children)**: a) Anemia: Hb < 11.5 g/dL; b) Microcytosis: MCV < 80fL, normocytosis: 80fL ≤ MCV ≤ 100 fL and macrocytosis: MCV ≤ 100fL; c) Normochromia: 32% ≤ MCHC ≤ 36% and hypochromia: MCHC < 32%.

According to the degree of severity of the anemia, we distinguish: a) Mild anemia, Hb level between 11 to 11.4 g/dL; b) Moderate anemia, Hb level between 8 and 10.9 g/dL; c) Severe anemia, Hb level below 8 g/dL.

**Group II (adults)**: a) Anemia: male, Hb < 13g/dL and anemia: female, Hb < 12g/dL; b) Microcytosis: MCV < 80fL, normocytosis: 80fL ≤ MCV ≤ 100 fL and macrocytosis: MCV ≤ 100fL; c) Normochromia: 32% ≤ MCHC ≤ 36% and hypochromia: MCHC < 32%.

**Group III (pregnant women)**: a) anemia: Hb < 11 g/dL; b) Microcytosis: MCV < 80 fL, normocytosis: 80 fL ≤ MCV ≤ 100 fL and macrocytosis: MCV ≤ 100 fL; c) Normochromia: 32% ≤ MCHC ≤ 36% and hypochromia: MCHC < 32%.

According to the degree of severity of the anemia, we distinguish: a) Mild anemia, Hb level between 10 to 10.9 g/dL; b) Moderate anemia, Hb level between 7 and 9.9 g/dL; c) Severe anemia, Hb level below 7 g/dL.

**Ethical Considerations**: written informed consent was obtained from each subject before enrollment in the survey in conformity with ethical standards. Written informed consent was also obtained from all children before registration via their guardian´s approval or parent for publishing the data and ensuring the correct completion of the questionnaire. Anonymity was guaranteed and a written declaration was also inserted in the preamble of the questionnaires providing additional information on the study's aim and the confidentiality of data related to the research. This study was approved by the health committee of the Provincial Hospital Center Mohammed VI of M´diq under number 1017.

**Statistical analysis**: for statistical analysis, data were entered and recorded on Excel 2013. The data then were analyzed using SPSS IBM-Statistics^®^ 20.0 software (Inc, Chicago, Il). The analysis was stratified by gender and age groups. Using descriptive statistics, all variables were compiled (numbers (N) and percentages (%)). The Chi-squared test and Kruskal-Wallis test were done for nonnormally distributed parameters with the Bonferroni method adjustment of p-value. Epi info 2000 software was used to determine the anthropometric indicators (Z-scores) by the “NUTSTAT” application. The Odds Ratios (OR) and p-value were generated using bivariate and multivariate logistic regression models to identify the association between sociodemographic factors and anemia among all tested groups. In multivariable logistic regression analysis variables with p-value < 0.05 was taken as statistically significant.

## Results

**Sociodemographic characteristics of the study population**: our reference population was composed of 1285 individuals divided into three groups, the socio-demographic characteristics of the study population are represented in Table 1:

**Table 1:** sociodemographic characteristics of the study population

Group I: School-age children				Group II: Adults				Group III: Pregnant women			
Features		N	%	Features		N	%	Features		N	%
**Gender**	**Female**	236	61.3	**Gender**	**Female**	408	79.5	**Trimester**	**First trimester**	157	40.6
	**Male**	149	38.7		**Male**	105	20.5		**second trimester**	167	43.2
	**Total**	587	100		**Total**	513	100		**Third trimester**	63	16.3
**Age (years)**	**5 à 7**	177	46	**Age (years)**	**16 à 30**	141	27.5	**Age (years)**	**< à 25**	132	34.1
	**8 à 11**	208	54		**31 to 45**	158	30.8		**25 to 35**	147	38.0
	**Total**	385	100		**> à 45**	214	41.7		**> à 35**	108	27.9
**Mother's level of education**	**Analphabetic**	245	63.6	**Level of education**	**Analphabetic**	292	56.9	**Level of education**	**Analphabetic**	153	39.5
	**Primary and higher**	140	36.4		**Primary and higher**	221	43.1		**Primary and higher**	234	60.5
**Parent's socioeconomic level**	**Low**	164	42.6	**Socio-economic level**	**Low**	232	45.2	**Socio-economic level**	**Low**	172	44.4
	**Medium**	216	56.1		**Medium**	274	53.4		**Medium**	210	54.3
	**High**	5	1.3		**High**	7	1.4		**High**	5	1.3
**Place of residence**	**Urban**	354	91.9	**Place of residence**	**Urban**	469	91.4	**Place of residence**	**Urban**	246	63.6
	**Rural**	31	8.1		**Rural**	44	8.6		**Rural**	141	36.4

**Group I**: 236 children (or 61.3%) were male compared to 149 children (or 38.7%) who were female, with a sex ratio of 1.58. In 63.6% of cases, the children's mothers were not in school. 56.1% of the children's parents belonged to a middle socioeconomic level. The majority of the children in the study were from urban areas (91.9%).

**Group II**: 408 adults (79.5%) were male compared to 105 adults (20.5%) who were female, with a sex ratio of 3.88. 214 adults (41.7%) were older than 45 years. More than half of the adults were illiterate (56.9%) and belonged to a middle socioeconomic level (53.4%).

**Group III**: 167 pregnant women, or 43.2%, were in the 2^nd^ trimester of pregnancy. The age group of 25 to 35 years was the most dominant, comprising 38% of the sample. The rate of pregnant women with primary education and above was 60.5% and only 1.3% of these women belonged to a high socioeconomic level. Moreover, 36.4% of the pregnant women lived in rural areas.

### Results of anemia prevalence and distribution of the study population by sex, age groups, and anemia intensity (Table 2)

This study revealed that 35.7% (Table 2) of pregnant women in the study area were anemic. This prevalence differed according to age groups, with the <25 age group having the highest prevalence among pregnant women in our study followed by the age group >35 years with a prevalence of 33.3% and the age group 25-35 years had a lower prevalence in our study population with a percentage of 31.2%. The prevalence of anemia was 60.9% in the 2^nd^ trimester, 29% in the 3^rd^ trimester, and 10.1% in the 1^st^ trimester of pregnancy in the northern region of Morocco. Mild anemia was the most common type, affecting 64.5% of pregnant women, followed by moderate anemia in 33.3% of participants. Severe anemia was less common, affecting only 2.2% of pregnant women in the present study.

**Table 2:** prevalence of anemia and distribution of the study population by gender, age groups and anemia intensity

Group I: school -age children	Boys	Boys	Boys		Girls	Girls		Girls	Girls
	Parameters		N	%	Parameters			N	%
	Hb (g/dL)	Hb≥11.5	100	67.1	Hb (g/dL)		Hb≥11.5	157	66.5
		Hb<11.5	49	32.9			Hb<11.5	79	33.5
	Age groups (years)	5 à 7	21	42.9	Age groups (years)		5 à 7	38	48.1
	Hb<11.5 g/dL	8 à 11	28	57.1	Hb<11.5 g/dL		8 to 11	41	51.9
	Intensity of Anemia	11 to 11.4	8	16.3	Intensity of Anemia		11 à 11.4	14	17.7
	Hb(g/dL)	8 à 10.9	41	83.7	Hb(g/dL)		8 à 10.9	63	79.7
			0	0				2	2.5
	Men				Women				
**Group II: Adults**	Parameters	< à 8	N	%	Parameters		< à 8	N	%
	Hb (g/dL)	Hb≥13	70	66.7	Hb (g/dL)		Hb≥12	217	53.2
		Hb<13	35	33.3			Hb<12	191	46.8
	Age range (years)	16-30	5	14.3	Age range (years)		16-30	56	29.3
	Hb<13 g/dL	31-45	4	11.4	Hb<12 g/dL		31-45	60	31 .4
		>45	26	74.3			> 45	75	39.3
	Intensity of Anemia	11 à 12.9	23	65.7	Intensity of Anemia		11 to 11.9	92	48.2
	Hb(g/dL)	8 à 10.9	10	28.6	Hb(g/dL)		8 à 10.9	86	45
			2	5.7			< à 8	13	6.8
**Group III : Pregnant women**	Parameters	< à 8	N		(%)				
	Hb (g/dL)	Hb≥11	249		64.3				
		Hb<11	138		35.7				
	Age groups (years)	<25	49		35.5				
	Hb<11 g/dL	25-35	43		31.2				
		>35	46		33.3				
	Intensity of anemia	10 to 10.9	89		64.5				
	Hb(g/dL)	7 to 9.9	46		33.3				
		< to 7	3		2.2				
	Trimester	First	14		10.1				
	Hb<11 g/dL	Second	86		60.9				
		Third	40		29				
									

The prevalence of anemic children in our study population was 32.9% and 33.5% in boys and girls respectively. Indeed, among school-aged children [8-11] years in the northern region of Morocco (M'dieq, Fnideq-Martil) the prevalence of anemia was 57.1% and 51.9% among boys and girls respectively. In the group of school children, mild anemia was observed in boys and girls 16.3% and 17.7%, respectively. In the adult group, the anemia in females was higher (46.8%) than in males (33.3%). In the age group [31-45] the prevalence of anemia (Table 2) was 31.4% in women and 11.4% in men. Concerning the oldest age group >45 years, the prevalence of anemia in all men was 74.3% and 39.3% in women. In this study, mild anemia was observed in 65.7% of men and 48.2% of women. Moderate anemia was present in 45% of women and 28.6% of men and severe anemia affected 6.8% of women and 5.7% of men.

**Results of anemia prevalence in school-aged children by anthropometric characteristics and dietary status**: Table 3 presents the anthropometric indices (height for age (H/A), weight for age (W/A), and body mass index (BMI) for age (BMI/age) z scores) and dietary status (frequency of consumption of meat products, vegetables, and fruits) depending on the anemic and non-anemic (values of Hb) in schoolchildren studied. However, when the anemic status is examined as a risk factor for stunting and underweight, the Odds Ratio (OR) for height-for-age expressed as a Z-score is 5.00. Children with insufficient stature are at high risk for anemia compared to children of normal stature (p<0.001). As for weight for age, the OR was 4.32. A significant difference between underweight and anemia is thus revealed by these results (p<0.001). Thinness, as assessed by BMI, did not appear to be related to anemia in this study (OR = 0.96). The average frequency of consumption of meat products is 1.8 times per week. A frequency less than 1.5 times/week exposes schoolchildren to a double risk of being anemic compared to a child consuming meat at least twice a week, the prevalence is 38.9% in the former against 29.9% in those who consume meat products more frequently. The same degree of risk affects children consuming less than 1.5 times weekly of vegetables and fruits. But with no significant relationship between anemia prevalence and dietary diversity score, the OR was 1.47 (p=0.086).

**Table 3:** prevalence of anemia in school-aged children according to anthropometric characteristics and dietary status

Parameters	Hb<11.5 g/dL		Hb≥11.5 g/dL		Odds Ratio	P value
	Number	%	Number	%		
Anemia and stunting: Z-score Size/Age (S/A)						
Z< -2	59	60.8	38	39.2	5	0.000 a
Z ≥ -2	69	24	219	76		
anemia and underweight: Z-score Weight/age (W/A)						
Z < -2	40	61.5	25	38.5	4.32	0.000 a
Z ≥ -2	88	27.5	232	72.5		
Anemia and thinness: Z-score Body mass index/age (BMI/A)						
Z < -2	12	32.4	25	67.6	0.96	0.912 b
Z ≥ -2	116	33.3	232	66.7		
Frequency of meat product						
< 1.5/week	56	38.9	88	61.1	1.51	0.070 b
> 2 times/week	72	29.9	169	70.1		
Frequency of vegetables/fruits						
< 1.5/week	60	38.2	97	61.8	1.47	0.086 b
> 2 times/week	68	29.8	160	70.2		

Statistical comparison: Chi-squared test and Kruskal-Wallis test were done for nonnormally distributed parameters with the Bonferroni method adjustment of p-value. The Odds Ratios (OR) and p-value were determined using bivariate and multivariate logistic regression models to identify the association between sociodemographic factors and anemia among all tested groups. In multivariable logistic regression analysis variables with p-value < 0.05 was taken as statistically significant. a) statistically significant; b) statistically non-significant.

### Results of the distribution of the study population by type of anemia (Table 4)

Distribution of the population studied by type of anemia (Table 4): 40.6% of school-aged children were classified as having microcytic hypochromic anemia, 48.7% of adults, and 43.5% of pregnant women as having had the same type of anemia. Secondly, normocytic hypochromic anemia was observed in 11.7% of preschool children, 6.6% of adults, and 2.9% of pregnant women. In this investigation, macrocytic hypochromic anemia was only found in the adult group with a percentage of 3.5%. Microcytic normochromic anemia was identified in 9.4% of school children, 1.8% of children, and 10.1% of pregnant women. The highest type of anemia in this study was normocytic normochromic anemia with a percentage of 34.9% in school children, 37.2% in the adult group, and 38.4% in pregnant women. Normochromic macrocytic anemia was detected in 2.3% of children, 2.2% of adults, and 5.1% of pregnant women.

**Table 4:** distribution of the study population by type of anemia

Study population	Group I: School-age children		Group II: Adults		Group III: Pregnant women	
Type of anemia	Headcount	%	Headcount	%	Headcount	%
Microcytic hypochromic anemia	52	40.6	110	48.7	60	43.5
Normocytic hypochromic anemia	15	11.7	15	6.6	4	2.9
Macrocytic hypochromic anemia	00	00	8	3.5	0	00
Microcytic normochromic anemia	12	9.4	4	1.8	14	10.1
Normocytic normochromic anemia	46	34.9	84	37.2	53	38.4
Macrocytic normochromic anemia	3	2.3	5	2.2	7	5.1
**Total**	128	100	226	100	138	100

### Results of sociodemographic factors associated with anemia (Table 5)

The parents of the children included in this study are illiterate with a percentage of 63.6%, the socio-economic level of the parents was an average of 56.1%, and the urban environment was 91.9%. The education level of parents was significantly associated with anemia (p<0.05), while the place of residence, age ranges, and socioeconomic status showed no association with anemia in school children, the p-values were 0.190, 0.139, and 0.974, respectively. The group of adults consisted of 56.9% of illiterates, a socioeconomic level of 53.6% having an average level and coming in most of the urban environment (91.4%). The socioeconomic level of adults was significantly associated with anemia (p<0.05) followed by the place of residence (p=0.088), whereas age groups (p=0.848) and education level (p=0.10) did not relate to anemia among adults. The group of pregnant women was 60.5% primary and above, the socio-economic level was average of 54.3% and the place of origin was rural (63.6%). Education level was significantly associated with anemia (p<0.05) followed by age groups (p=0.082), whereas the place of residence (p=0.779) and socioeconomic level (p=0.156) were not related to anemia in pregnancies.

**Table 5:** sociodemographic factors associated with anemia

Group I: School-age children					Group II: Adults					Group III: Pregnant women				
**Features**		Total N(%)	Anemia %	P-value	**Features**		Total N(%)	Anemia %	P value	**Features**		Total N(%)	Anemia %	P-value
**Age (years)**	5 to 7	177(46.0)	15.3	0.974 b	**Age (years)**	16 à 30	141(27.5)	11.9	0.848 b	**Age (years)**	< à 25	132(34.1)	12.7	0.082 b
	8 à 11	208(54.0)	17.3			31 to 45	158(30.8)	12.5			25 à 35	147(38.0)	11.1	
	Total	385(100)	32.6			>à 45	214(41.5)	19.7			>à 35	108(27.9)	11.9	
**Parents' level of education**	Analphabetic	245(63.6)	23.0	0.018 b	**Level of education**	Analphabetic	292(56.9)	27.9	0.10 b	**Level of education**	Analphabetic	153(39.5)	16.8	0.023 a
	Primary and higher	140(36.4)	9.4			Primary and higher	221(43.1)	16.2			Primary and higher	234(60.5)	18.9	
**Parent's socioeconic level**	Low	164(42.6)	15.3	0.139 b	**Socio-economic level**	Low	232(45.2)	22.8	0.013 b	**Socio-economic level**	Low	172(44.4)	18.1	0.156 b
	Medium	216(56.1)	17.7			Medium	274(53.6)	21.1			Medium	210(54.3)	17.3	
	High	5(1.3)	0.3			High	7(1.4)	0.2			High	5(1.3)	0.3	
**Place of residence**	Urban	354(91.9)	31.4	0.190 b	**Place of residence**	Urban	469(91.4)	41.3	0.088 b	**Place of residence**	Rural	246(63.6)	23.0	0.779 b
	Rural	31(8.1)	1.8			Rural	44(8.6)	2.7			Urban	141(35.7)	12.7	

Statistical comparison: Chi-squared test and Kruskal-Wallis test were done for nonnormally distributed parameters. p-value was determined using a multivariate logistic regression model to identify the association between sociodemographic factors and anemia among all tested groups with p-value < 0.05 taken as statistically significant. a statistically significant. b statistically non-significant.

## Discussion

The present study aimed to estimate the prevalence and determine the various factors associated with anemia in a population consisting of adults, pregnant women, and school children living in the M'diq-Fnideq prefecture, northwestern Morocco.

**Prevalence of anemia in pregnant women**: pregnant women are one of the vulnerable groups in a population to develop anemia, especially in developing countries [6]. This study revealed that 35.7% (Table 2) of pregnant women in the study area were anemic. According to the WHO classification of public health importance of anemia, the magnitude indicates that there is moderate public health importance of anemia among pregnant women in the study area [7]. The prevalence of anemia obtained in this study is almost consistent with other studies conducted among pregnant women attending antenatal clinics in Sidama [8], West [9], and Northern Nigeria [10], with a prevalence of 31.6%, 36.6%, and 30%, respectively. The result of this study was much higher than other studies conducted among pregnant women attending Adigrat General Hospital with a prevalence of 7.9% [11], Debre Berhan, 9.7% [12], Sudan, 10% [13], Addis Ababa, 11.6% [14] and Iran, 13.6% [15]. The results of the present study were much lower than the previous studies conducted among pregnant women attending antenatal clinics in North Bengal [16], Udupi district [6], Bangladesh [17] with a prevalence of 82%, 50.1%, and 73%, respectively. The difference could be due to geographical variation, differences in socioeconomic status, and dietary habits of the study participants [14].

This prevalence differs according to age groups. In fact, the age group <25 years has the highest prevalence among pregnant women in our study followed by the age group >35 years with a prevalence of 33.3% and the age group 25-35 years had a lower prevalence in our study population with a percentage of 31.2%. In addition, anemia prevalence increased according to the term of pregnancy. Pregnant women in the 2^nd^ trimester had a prevalence of 60.9%, the 3^rd^ trimester of pregnancy had a prevalence of 29% and the 1^st^ trimester of pregnancy among pregnant women in the northern region of Morocco was 10.1%. In this study, the majority of cases of anemia having mild anemia (64.5%) followed by (33.3%) of pregnant women having moderate anemia, regarding severe anemia among the participants in the present study was lower (2.2%), which was similar to the study of Suryanarayana R *et al*. and Kapil and Sareen (1.6%) [18,19]. However, other studies have reported a higher prevalence of Totega (13.1%) and Gautam *et al*. (22.8%) [20,21]. The high prevalence of anemia may be attributed to low dietary iron and folic acid intake, poor iron bioavailability, or chronic blood loss due to infections.

**Prevalence of anemia in school children**: the prevalence of anemic school children in our study population was 32.9% and 33.5% in boys and girls, respectively (Table 2). These results are consistent with the results of a meta-analysis that showed no difference according to gender [22]. However, a higher prevalence was observed in a population-based study by Rivera *et al*. which included preschool children in Cuba, who reported a prevalence of 55.6% [23]. Sanabria reported a prevalence of 52% in children under 5 years of age in a referral hospital in Paraguay [24]. The influence of gender on anemia yields conflicting results. This study and others found no association between anemia and gender [25-27], while other studies reported that anemia is more common in boys [28]. This may be due to the faster growth of preschool boys compared to girls, which results in increased food intake. Indeed, among school-aged children [8-11] years in the northern region of Morocco (M'dieq, Fnideq-Martil) the prevalence of anemia was 57.1% and 51.9% in boys and girls respectively. In fact, we observed no difference between girls aged 8-11 years and boys at these ages, these results are in agreement with that of Iglesias Vázquez L *et al*. [22]. In the group of school children, mild anemia was observed in boys and girls at 16.3% and 17.7%, respectively (Table 2). These results did not show differences according to the intensity of anemia and gender of school children. These outcomes are in agreement with those recently obtained by Irisarri-Gutiérrez M *et al*. 2022 in anemic schoolchildren who did not show significant differences by sex or age group, in contrast to Mupfasoni D *et al*. [29], who found that boys aged 5-7 years were more likely to be anemic. Moderate anemia was found in almost all children at preschool age with a percentage of 83.7% in boys and 79.7% in girls (Table 2). Among the investigated variables, age had a considerable correlation with anemia. In this research, we found that the hemoglobin concentration increased with age. Moderate anemia was identified in almost all children at preschool age with a percentage of 83.7% in boys and 79.7% in girls.

### Prevalence of anemia in adults

In the adult group, anemia was higher in women (46.8%) than in men (33.3%) (Table 2), with similar results among adolescents in India: 37% anemic adolescents. Considering that anemia is a consequence occurring at a later stage of iron deficiency, the prevalence of anemia in these adolescent girls is 39%, of which 37% are due to iron deficiency, which should be considered significant and requires attention [30]. The UNICEF/WHO Joint Committee on Health Policy (JCHP) recommends iron supplementation for all women aged 10-49 years in countries where more than 30% of the population is anemic [31]. In the group of patients in the age range [32-46], the prevalence of anemia (Table 2) is higher in women (31.4%) versus in men (11.4%). These findings are consistent with a study showing that the prevalence of anemia was higher in women under 65 years of age than in men in the same age group (53% versus 44%, respectively). This divergence may be partly attributable to the menstrual blood loss component in the first subset of patients. Overall, these observations are in harmony with previous research on the incidence of anemia in this age category [34,38,41,43].

Regarding the oldest age group age >45 years, the prevalence of anemia in all men was higher (74.3%) than in women (39.3%) (Table 2). However, Chueh *et al*. suggest that the prevalence of anemia was higher in the population aged ≥ 65 years than in the younger population. Anemia was also more common in women than in men, but this difference was not significant in those aged > 85 years [40]. Indeed, the occurrence of anemia in the elderly is not fully understood. Hemoglobin levels in the elderly population are reportedly lower than the reference values for other population groups. Some reports have concluded that this decrease in Hb levels may be one of the consequences of the normal aging process [35,36,38]. Nevertheless, several studies have shown that anemia in the elderly is related to the existence of underlying health problems, and thus to a high mortality and morbidity rate. In addition, it has been shown that most people with anemia suffer from nutritional insufficiency, although the cause of one-third of anemia cases remains unclear [33,45]. This prevalence was higher compared to other populations, in Turkey, the prevalence of anemia in the elderly was 7.3%, with the prevalence of anemia in men (9.2%) being higher than in women (5.3%) [47]. In Pakistan, 31.0% of the elderly were anemic [42]. In the United States, 11.0% of the men and 10.0% of the women aged 65 years and older had anemia and this figure doubled in people over 85 years of age [37]. In England, the prevalence of anemia was 5.2% [32], which was lower than the present study.

In this investigation, mild anemia was observed in 65.7% of men and 48.2% of women (Table 2), moderate anemia was present in 45% of women and 28.6% of men (Table 2), and severe anemia in 6.8% of women and 5.7% of men (Table 2) Mild anemia was the most common type in the present study, and moderate anemia was more common than severe anemia. This result was similar to a study from Nigeria that reported that most of the observed cases of anemia were mild in severity and 14.9% of all anemic patients had a severe form of anemia [44]. Other studies have reported a prevalence of anemia of 11.8%, 5.4%, and 8-44% in adults, which is lower than our results [48,49]. The present study showed that mild anemia is highly prevalent among adults in our population.

### Distribution of the study population by type of anemia

Analysis of the study population by type of anemia (Table 4) showed that microcytic hypochromic anemia was detected in 40.6% of schoolchildren, 48.7% of adults, and 43.5% of pregnant women. Following this, normocytic hypochromic anemia was noted in 11.7% of preschool children, 6.6% of adults, and 2.9% of pregnant women. In this survey, macrocytic hypochromic anemia was only seen in the adult group with a percentage of 3.5%. Microcytic normochromic anemia was encountered in 9.4% of school children, 1.8% of adults and 10.1% of pregnant women. The most frequent type of anemia in this research was normocytic normochromic anemia with a percentage of 34.9% in school children, 37.2% in the adult group and 38.4% in pregnant women. The macrocytic normochromic anemia type was diagnosed in 2.3% of children, 2.2% of adults and 5.1% of pregnant women. As described in the current work, the predominant form of anemia was microcytic hypochromic anemia with a percentage exceeding 40%. Comparable evidence has been reported in other previous studies where the substantial predominance of anemia was microcytic [35,50].

However, studies have suggested that the main anemia was normocytic normochromic with a prevalence of 46% normocytic normochromic anemia as the most common type of anemia [51]. A study in Italy and Brazil reported 88% and 72.3% of cases of normocytic anemia [44,52], but this type of anemia represents the second most common anemia in this study with a percentage of 37.2%. The present study showed 6.6% of normocytic hypochromic anemia, macrocytic hypochromic anemia (3.5%), and macrocytic normochromic anemia (2.2%). This shows that the causes of anemia in the elderly and the young are distinct. In the Third National Health and Nutrition Examination Survey, NHANES III, one-third of the population over 65 years of age had anemia caused by dietary deficiencies, with the remainder having anemia caused by chronic disease or unexplained anemia [35]. This survey also found that younger women had a higher incidence of iron deficiency than women over 50 years of age and men of the same age, which may explain why women of childbearing age have a higher incidence of microcytic anemia [53].

### Associated factors among anemic subjects (Table 3 and Table 5)

In school-age children, Hb concentration can be partially explained by the following factors, the educational (p=0.018) and socioeconomic level of parents but also the frequency of consumption of meat, vegetable and. Although weak correlations have been observed, it seems that further approaches to elucidate this are necessary. Children with illiterate parents are the most affected by anemia with a prevalence of 75% and only 20% for educated parents. Several studies have proven that the education of parents and especially mothers have an impact on the children's health [54,55]. Indeed, the experience acquired by the mother could protect the child from anemia through her functional literacy and cognitive skills. This protection is achieved by providing a stimulating, adequate, and nutritionally balanced environment [55]. The prevalence of anemia was found in 33% of children among parents of low/middle socioeconomic status and it was positively associated with socioeconomic level (p<0.05). These findings are concordant with a Brazilian study conducted on a sample of 603 preschool children in which inadequate socioeconomic conditions at home were significantly associated with an increased risk of anemia with odds of 2.09 (95% CI: 1.22-3.60) in school children presenting anemia [56]. In our context, living in urban or rural settings did not appear to be an associated factor with anemia (p=0.190) in school-age children, however, Ncogo P *et al*. in their study performed on 1436 children from Equatorial Guinea, found that area of residence had an impact on the occurrence of anemia, the prevalence of anemia was higher among children living in rural than urban settings (p<0.001) [57]. In addition, children with stunting are at high risk for anemia compared to children of normal stature (p<0.001). As for weight for age, the OR was 4.32. A significant difference between underweight and anemia is thus revealed by these results (p<0.001). These results are consistent with those reported in the study carried out by Aboussaleh Y *et al*. in which 65% of anemic school children have stunted growth with an Odds Ratio (OR) of 4.64 and 63.6% were underweight [58]. Based on dietary status, our results showed that a frequency less than 1.5 times/week exposes the school children to a double risk of being anemic compared to a child consuming meat, vegetables, and fruits at least twice a week (Table 3), these observations corroborate with those found in the study conducted by Yessoufou AG *et al*. in which, among 251 malnourished children, the prevalence of anemia was considerable 41.43% [59]. Similarly, Caicedo-Gallardo JD and colleagues indicated in their cross-sectional study that the prevalences of chronic malnutrition and anemia in the children were 12.8% and 15.0% respectively and they indicated that the association between mothers' social status and the nutritional condition of their children in rural settings appears to be significantly related [60]. Also, using a multi-causal approach in children under 5 years of age from Ecuador, the prevalence of anemia was 16.98% (n = 54) and 12.42% (n = 39) for chronic malnutrition and it noted a significant association between anemia, chronic malnutrition, and socio-economic situation [61]. Moreover, Yang W. *et al*. reported in their survey that 35.12% of infants in rural Shaanxi suffered from anemia, and the malnutrition prevalence rates were 32.14% for underweight, 11.31% for wasting, and 39.58% for stunting. Anemia was found to be significantly correlated with malnutrition (stunting, OR: 1.65, 95% CI: 1.05-2.61; wasting, OR: 2.89, 95% CI: 1.45-5.76; underweight, OR: 2.42, 95% CI: 1.50-3.88) [62]. As evidenced in several investigations [54,61,63-65], the association between anemia and malnutrition in its different aspects is revealed by stunting and being underweight. This is possible through high immunity and inhibition of growth rates of bacteria and parasites that require iron for their development, also, anemia and iron deficiency are associated with impaired neurocognitive and psychomotor development in children. However, this hypothesis cannot be confirmed as the iron specificity of the anemia is not studied in this case and the dietary intake pattern followed in our study does not explain the nutritional status of this study group, nor the occurrence of anemia.

In pregnant women, the prevalence of anemia was 35.7% which was significantly associated with educational level (p<0.05) than socioeconomic level (p=0.156), the environment of residence (p=0.779), and age class (p=0.082). Whereas, in a study established by Lebso M *et al*. which involved 507 pregnant women living in Southern Ethiopia, the reported prevalence of anemia was 23.2% (95% CI: 19.5%-26.9%). Low socioeconomic status was among factors related to anemia (OR = 2.03, 95% CI: 1.11-3.69) as well as second trimester (OR = 3.09, 95% CI: 1.41-6.79) and third trimester (OR = 3.68, 95% CI: 1.67-8.08) [66]. Similarly, in a retrospective study of 44002 pregnant women, the prevalence of anemia was 23.5% in which anemia was significantly related to rural residence with an OR of 1.308 which is not consistent with the results obtained in our study (p=0.779) [67]. Indeed, the main cause of anemia in pregnant women is iron deficiency as shown in the study by Zhang N *et al*. carried out on 789 pregnant women, the prevalence of iron deficiency anemia was 39.8% and they hypothesized that moderate physical activity, high frequency of iron-rich food intake could prevent against iron deficiency anemia during pregnancy [68]. Also, in the national survey by Tan, J *et al*. conducted on 12403 pregnant women, 19.8% of women were identified with anemia and 13.9% were diagnosed with iron deficiency anemia and it was associated with non-local residents and multiple gestations [69].

In the group of adults, anemia was significantly associated with low socioeconomic levels (p<0.05) and showed no association with age class (p=0.848), level of education (p=0.10), and place of residence (p=0.088). However, the investigation by Zegeye B *et al*. performed on 6809 women aged between 15 and 49 years indicated that anemia was related to the current employment situation (OR = 0.77, 95% CI; 0.61-0.96) and living region (Douala region OR = 2.65, 95% CI; 1.61-4.36, North-West region OR = 0.53, 95% CI; 0.28-0.99) [70]. Furthermore, another investigation designed by Pratima V *et al*. was performed to determine the frequency of anemia and to highlight associated factors among 609 healthy adults from north India. The results showed that the frequency of anemia in males and females was 70.1% and 53.2%, respectively. Anemia was significantly related to lower sociodemographic (p<0.0001) and socioeconomic levels (p<0.0089) and sedentary lifestyles (p<0.0001) in both females and males [71]. Indeed, in adult subjects, anemia could be a health risk factor and lead to cardiovascular complications and sometimes early death. In addition, it induces tiredness and has a significant negative effect on physical and cognitive abilities as well as on the quality of lifestyle [72].

## Conclusion

Anemia is a common multifactorial disorder affecting children, adults, the elderly, and pregnant women, and is a major public health challenge in Morocco. It is a global health issue that has significant impacts on human health and socio-economic, socio-demographic, cognitive, and psychomotor development, affecting both developed and developing countries. The prevalence of anemia in our sample of school children, adults, and pregnant women was 32.6%, 44.10%, and 35.7%, respectively, indicating a significant health concern in the surveyed prefecture. In school-aged children, anemia is an indicator of nutritional and health deterioration and has been associated with stunting and being underweight. Parental education and socioeconomic status are considered protective factors for children. Among the adults tested, anemic women and men had similar frequencies across different age groups. The socioeconomic and educational levels of patients also had a significant effect on their health status. The most common type of anemia diagnosed was microcytic hypochromic anemia, which could be due to iron deficiency. In the sample of pregnant women, anemia was more common in women under 25 years and over 35 years of age. Microcytic hypochromic anemia was the most common type of anemia, followed by normocytic normochromic anemia and normocytic hypochromic anemia. As pregnancy progresses, the risk of anemia increases, particularly in the second and third trimesters. Therefore, extensive community surveys are necessary to investigate the origin and type of anemia and to assess additional health risk factors in all age groups and genders.

### What is known about this topic

- *Anemia represents a critical public health issue in Morocco with significant repercussions on human health, especially among pregnant women and children;*
- *Several factors, including socioeconomic status, educational level, and nutritional intake, can affect the occurrence of anemia;*
- *Health institutions in Morocco face the challenge of accurately assessing the prevalence of anemia and its associated factors in the population, which highlights the need for greater awareness and research in this area*.

### What this study adds

- *In the investigated area, anemia was more prevalent in our sample consisting of school children, adults and pregnant women, with 32.6%, 44.10% and 35.7%, respectively;*
- *School children from low socioeconomic backgrounds and whose parents are illiterate, as well as those who are malnourished, are at greater risk of anemia;*
- *The most common type of anemia diagnosed was microcytic hypochromic anemia*.
